# Key Processes of Silicon-On-Glass MEMS Fabrication Technology for Gyroscope Application

**DOI:** 10.3390/s18041240

**Published:** 2018-04-17

**Authors:** Zhibo Ma, Yinan Wang, Qiang Shen, Han Zhang, Xuetao Guo

**Affiliations:** 1 He Ministry of Education Key Lab of Micro/Nano Systems for Aerospace (Northwestern Polytechnical University), Ministry of Education, Xi’an 710072, China; wangyinanmic@outlook.com (Y.W.); shenq@nwpu.edu.cn (Q.S.); zhanghan016@126.com (H.Z.); uestc-gxt@foxmail.com (X.G.); 2 Shaan’xi Key Lab of MEMS/NEMS, Northwestern Polytechnical University, Xi’an 710072, China

**Keywords:** SOG, SOI, fabrication, CMP, gyroscope, MEMS

## Abstract

MEMS fabrication that is based on the silicon-on-glass (SOG) process requires many steps, including patterning, anodic bonding, deep reactive ion etching (DRIE), and chemical mechanical polishing (CMP). The effects of the process parameters of CMP and DRIE are investigated in this study. The process parameters of CMP, such as abrasive size, load pressure, and pH value of SF1 solution are examined to optimize the total thickness variation in the structure and the surface quality. The ratio of etching and passivation cycle time and the process pressure are also adjusted to achieve satisfactory performance during DRIE. The process is optimized to avoid neither the notching nor lag effects on the fabricated silicon structures. For demonstrating the capability of the modified CMP and DRIE processes, a z-axis micro gyroscope is fabricated that is based on the SOG process. Initial test results show that the average surface roughness of silicon is below 1.13 nm and the thickness of the silicon is measured to be 50 μm. All of the structures are well defined without the footing effect by the use of the modified DRIE process. The initial performance test results of the resonant frequency for the drive and sense modes are 4.048 and 4.076 kHz, respectively. The demands for this kind of SOG MEMS device can be fulfilled using the optimized process.

## 1. Introduction

Microstructures fabricated based on silicon-on-glass (SOG) are always defined by through-etching of a thin silicon substrate anodically bonded to glass substrate [1]. Although silicon-on-insulator (SOI) wafers and silicon epitaxy can provide a certain thickness of the device layer [2], these preparation methods of silicon layer thickness are relatively limited. Silicon is chosen as a capacitor structure material because of its excellent mechanical properties. However, during the SOI process, significant notching occurs in the SOI micro fabrication process after the active silicon is etched to the buried oxide layer; this phenomenon is due to the reaction ions that are accumulating on the oxide layer. The charging causes a potential difference in the trench, thereby deflecting the injecting ions to bombard the lower corner sidewall and initiate the notch [3]. Although the notching effect is used to dry release the suspended structures, the bottom of the structure is seriously over etched during dry processing, an effect that is not conducive to the performance of the device control and mass production. Pyrex glass is used as a substrate instead of silicon to reduce the RF losses through the substrate over the high-frequency range [4] and may cause the notching effect; however, MEMS researchers have developed several solutions to eliminate or restrict the notching effect, such as patterning a metal film on the glass or on the bottom surface of silicon [5,6,7,8]. Thus, the SOG process sequence is better for fabricating complex, multi-level MEMS devices.

The SOG process mainly includesthe wet etching or drying etching silicon, anodic bonding of silicon and glass, chemical mechanical polishing (CMP) the silicon to obtain the certain thickness, and Deep reactive-ion etching (DRIE) the silicon to form the designed structures. The DRIE and CMP are the most important two processes for the SOG process. 

DRIE is used to define the movable structures in an inductively coupled plasma etcher and it might result in many silicon structures showing high aspect ratio [9,10]. After the notching effect is eliminated, the lag effect may play a role in the dry silicon etching process. This effect causes etch uniformity and difficulty in controlling the etch endpoint and degrades the mechanical performance, thereby leading to device failure and footing effect. The CMP process is used to maintain the thickness and quality of the structures, and thus it maintains the surface quality for the next anodic bonding process (e.g., sandwich structures). CMP techniques permit obtaining the desired thickness and surface quality and to meet the application requirements for different MEMS devices [11,12]. However, the methods are limited by the high surface quality and the high total thickness variation, which should be addressed [13]. 

The effect of process parameters on the silicon removal rate in CMP of the SOG process and the lag effect reduction is investigated in this paper. The dependencies of pressure and different slurry dilution ratios on silicon removal rate are also explored to solve the surface damages that are induced by breakthrough and stress concentration. Numerous factors affect the DRIE experimental results [14,15,16]. Thus, the main parameters including the flowrate of SF6, C4F8, oxygen, coil power, and platen power are adjusted to reduce the lag effect. As a result, the ratio of etching and passivation cycle time and the process pressure play more important roles in the lag effect. The process is optimized to avoid the notching and lag effects on the fabricated silicon structures during DRIE.

For demonstrating the capability of the silicon removal in CMP and lag effect reduction, a z-axis micro gyroscope is fabricated by the modified SOG process. According to the initial test result, the average surface roughness of silicon is below 1.13 nm and the thickness of the silicon is measured to be 50 μm. The calculated of the equivalent angular rate is about 1.6‰°/s/√Hz. Allan variance measurements is implemented with ARW of 1.026°/√h. The demands for this kind of SOG MEMS device can be fulfilled by the use of the optimized CMP process.

## 2. Concept and Process Exploitation 

### 2.1. CMP Process for Silicon

#### 2.1.1. Details of the CMP Process

Typical CMP processes of silicon wafer often consist of lapping and polishing. Lapping is always used firstly to thin the silicon wafer quickly using large particle size Al2O3 powder. To reduce the damaged silicon layer, Al2O3 powder with different particle size is chosen. After that, polishing of the silicon wafer is employed to further improve the surface quality. Polishing always consists of prepolishing and final polishing. The main aim to prepolishing is to wipe out the damaged silicon layer and distortion layer, resulting from lapping processes, and to achieve ordinary flatness and a degree of finish. The aim of final polishing is to remove the damaged layer resulting from the prepolishing processes and to improve the total thickness variation (TTV) and roughness of silicon. TTV of silicon is one of the factors affecting the performance of the fabricated sensors. In fact, the roughness of the silicon surface is related to the success rate of the next anodic bonding process.

Many factors to influence the polishing quality, such as the concentration and abrasive size, flow of polishing slurry, load weight, and the rotational speed. The load weight and the rotational speed have the most significant influence on the surface roughness of silicon wafer. In fact, we observe that if the load weight and the rotational speed increases, the damaged layer and the surface roughness of the silicon becomes worsen, otherwise the lapping and polishing rate of the silicon will be slow. These two aspects will be investigated in the following part.

The adopted PM5 precision lapping polishing machine with an automatic plate flatness control is from Logitech Ltd. (Scotland, UK). The lapping process to thin the silicon wafer involves two main stages. In particular, 20 μm Al_2_O_3_ powder is used in the first stage, and 9 μm Al_2_O_3_ powder is used in the second stage. The lapping pad is a 30 cm-diameter cast iron lapping plate with radial grooves.

For the polishing process, 3 μm cerium oxide powder and 30 cm-diameter expanded polyurethane polishing plate are used for the first polishing. Cerium oxide is commonly used in CMP for silicon removal, because it is softer than silicon and it causes less damage to the silicon film than other particles [17,18]. At this stage, 3 μm cerium oxide powder and SF1 abrasive can be used at the same time to obtain high planarity and wafer uniformity. The plate speed is 70 rpm, and the load weight is 4000 g. Finally, the chemcloth and SF1 abrasive are employed to the final polishing. The dilution ratio of SF1 abrasive is one part Rodel 1520 to three parts de-ionized water (1:3). Other details of the process parameters on silicon bonded to glass removal in CMP are shown in Table 1, and the measured thickness is the average obtained from five measurements.

#### 2.1.2. The Results of the CMP Process

**Figure 1:**
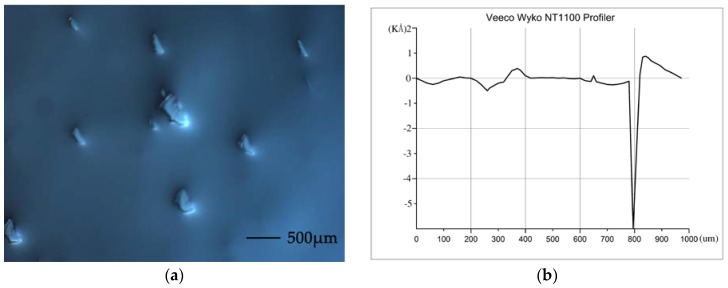
Photograph of the silicon surface and surface quality before CMP. (**a**) Silicon surface before CMP; (**b**) Measurement of the surface roughness.

The surface quality of the pre-bonded wafer is measured by BX51 microscope, which was acquired from Olympus Optical Co., Ltd. Tokyo, Japan. Some particles on the surface can be clearly observed at 200× magnification, as shown in Figure 1a. Figure 1b shows that the average surface roughness is 26.4 nm, as measured by Veeco Wyko NT1100 Profiler.

**Figure 2:**
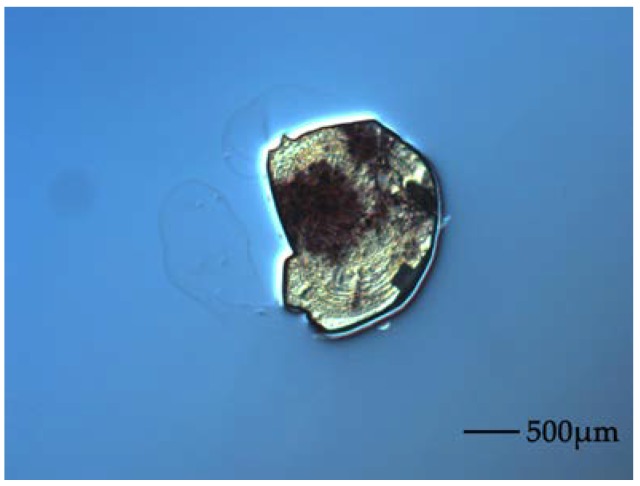
Photograph of the breakthrough on silicon surface.

The 9 μm Al_2_O_3_ powder is used before polishing silicon to decrease the damage of the silicon surface and to decrease the next polishing time. Meanwhile, the 3 μm cerium oxide powder plays a role in improving the total thickness variation of the wafer. However, several breakthroughs appear during pre-polishing the wafer using 3 μm cerium oxide powder, as shown in Figure 2. 

This phenomenon can be attributed to the poor alignment of the silicon and glass during anodic bonding that results in the silicon wafer over-hanging the glass slide. As a result, the silicon wafer becomes damaged as excessive stress occurs at these edges. Some silicon particles are also generated to damage the silicon surface during the polishing process. The underlying structures at localized points play a significant role with regard to surface quality. 

**Figure 3:**
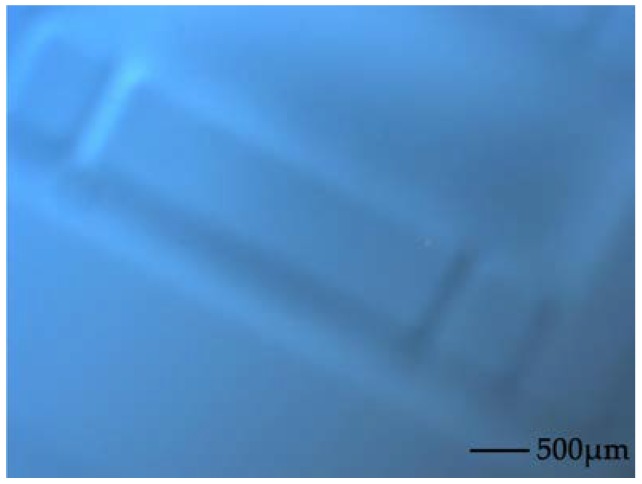
Photograph of the underlying structures that appeared during CMP.

Meanwhile, collapse of the underlying structures appears as the silicon thickness decreases, as shown in Figure 3. According to the detailed analysis, the collapse of silicon can be attributed not only to the bonding wax variation resulting in localized high and low points during anodic bonding techniques, but also to the higher weight that is loaded on the bonded silicon wafer during the CMP process.

To solve the abovementioned problems, wax uniformity is improved before the silicon and glass anodic bonded. At the same time, the load weight is reduced from 4000 g to 2500 g during the polishing process. Although the polishing rate slows down, the surface quality of the silicon improves and the collapse of silicon into the trenches also disappears. Finally, all of the damages can be significantly reduced, with consequent optimization of the CMP processes.

**Figure 4:**
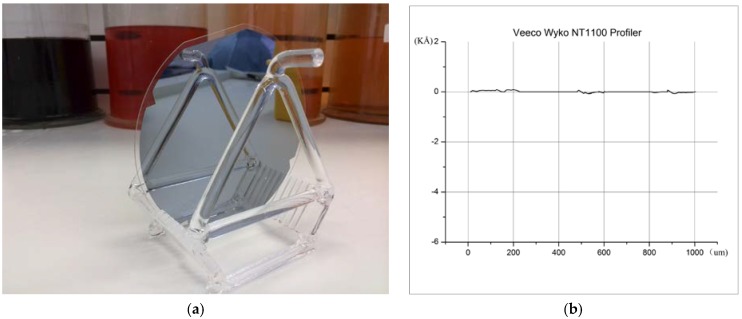
Top view of the final surface quality. (**a**) Final wafers with several test points; (**b**) Measurement of final surface quality

The final thickness of the silicon is measured to be 50 μm using a non-contact thickness gauge and the average surface roughness is 1.13 nm, as shown in Figure 4.

### 2.2. DRIE Process for Silicon

#### 2.2.1. Details of the DRIE Process

**Figure 5:**
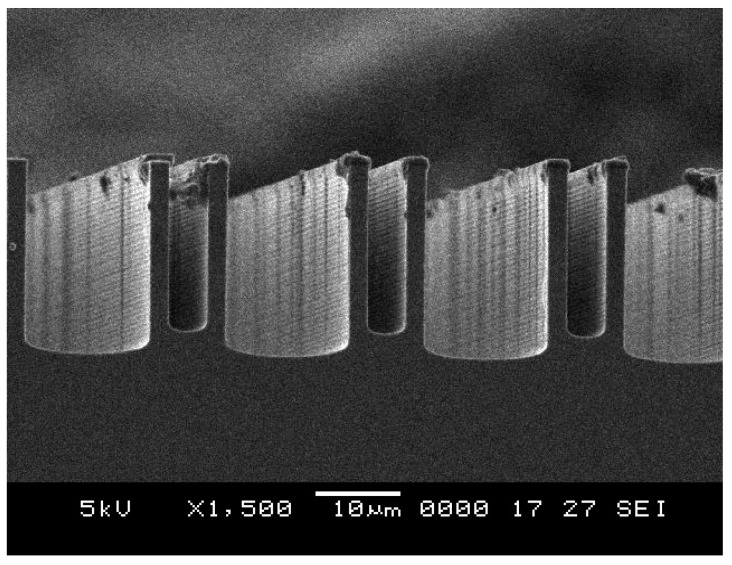
Scanning electron microscope (SEM) images of lag effect for unexpected Deep reactive-ion etching (DRIE) process results.

**Figure 6:**
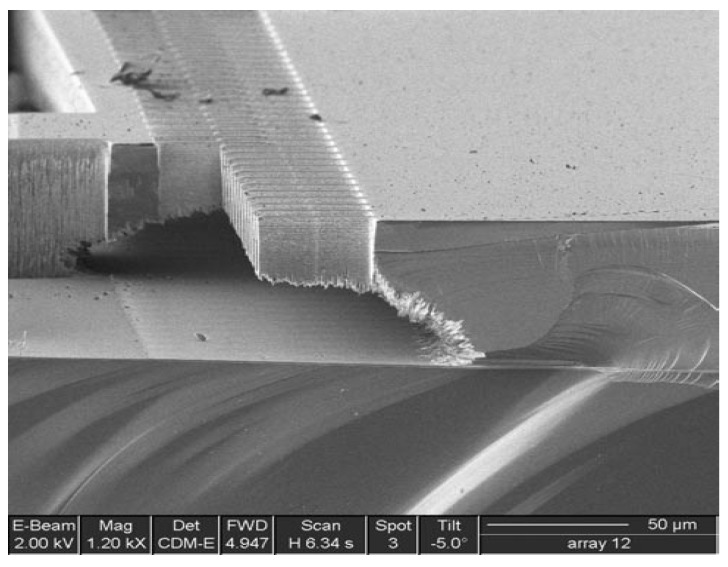
SEM images of footing effect for unexpected DRIE process results.

DRIE is the best choice for silicon dry etching with a high aspect ratio. Silicon etching with DRIE is limited mainly by the lag and footing effects, as shown in Figure 5 and Figure 6. The lag effect occurs due to the difference in the etch rate between the wide trench and narrow trench. In general, the etch rate is much higher for the wide trench than for the narrow one. 

The etch rate variation in the lag effect becomes essential for the through-etching of silicon or the active silicon of SOI wafers. After the wide trench is etched, an undercutting of the silicon occurs owing to the accumulation of positive charges in the insulator surface, such as the buried oxide or the Pyrex glass substrate. As a result, the anisotropic nature of DRIE is destroyed. The said effect is called notching or footing, and it causes significant destruction on the etched microstructures [19]. Figure 5 shows the scanning electron microscope (SEM) images of unexpected DRIE process results with the mechanism of lag effects, and Figure 6 shows the footing effect resulting in destruction at the bottom of a comb electrode array.

The lag effect not only results in etch uniformity and difficulty in controlling etch endpoint, but it also degrades the mechanical performance, thereby leading to device failure and footing effect. Thus, the lag effect should be primarily controlled. Although the methods of metal shield laid on the glass substrate under suspended silicon are proposed to decrease the damage that is caused by the lag and footing effects, the ions that are passing through the fast-etched trenches are collected by the shield, and the glass surface remains unchanged. Therefore, no significant damage is observed in the etched silicon during excessive etch periods [20].

The silicon structures with high verticality and high aspect ratio exhibit high performance and are thus widely used in the field of MEMS sensors and actuators. However, as described in [21], numerous factors affect the morphology of silicon structures during the DRIE experiment [22,23], such as the flowrate of SF_6_, C_4_F_8_, oxygen, coil power, and platen power.

According to the dry etching process in the MEMS fabrication laboratory, the ratio of etching and passivation cycle time and the process pressure play important roles in the lag effect. To verify this viewpoint, the above mentioned parameters are investigated, while the other parameters remain constant (Table 2). These parameters are adjusted following the literature in [21] to improve the silicon etching rate. For test recipes a, b, and c, the gas flows of SF_6_, O_2_, and C_4_F_8_ are 180, 20, and 120 sccm, respectively. The coil power is set to 800 W, and the platen power is set to 20 W.

**Figure 7:**
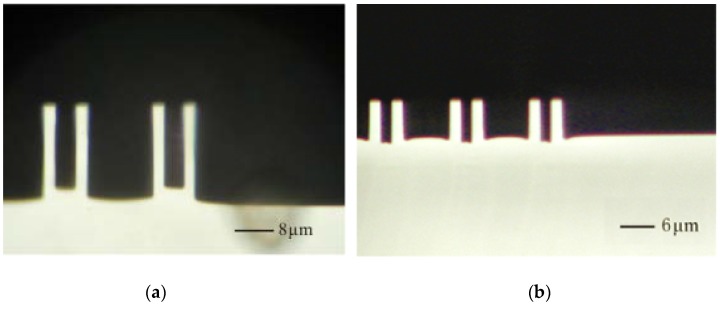
Cross-section views of DRIE results. All of the geometric parameters of the test structure are identical. (**a**) Lag effect; (**b**) Anti-lag effect.

Recipe a is first tested, in which the etching and the passivation times are 6 s and 5 s, respectively; the ratio of etching and passivation times is 1:2. Moreover, the process pressure is 43 mTorr. The cross-section view of the fabricated structures is shown in Figure 7a. The average etching rate is obtained as 4.67 μm/min. Furthermore, the wide trench is etched much faster than the narrow trench, and the lag effect appears clearly. This phenomenon can be serious with the increase in etching time.

In accordance with recipe a, the etching and passivation times are adjusted to 8 and 10 s. The process pressure is also adjusted to 35 mTorr (recipe b). Thereafter, the wide trench is etched lower than the narrow trench, and the lag effect produces better results. A different regime, called the anti-lag effect, is obtained and shown in Figure 7b.

As exhibited by the experiments on recipes a and b, the lag effect can be reduced by adjusting the relative duration of etch time against the passivation time and by reducing the process pressure. Thus, the footing effect can be conquered easily without lag effect, especially for the SOG or SOI process. Thus, optimal process parameters possibly exist between recipes a and b. 

#### 2.2.2. The Results of the DRIE Process

By continuously adjusting the process pressure, the optimal process pressure is obtained at 30 mTorr. The other suitable parameters are shown in Table 3.

**Figure 8:**
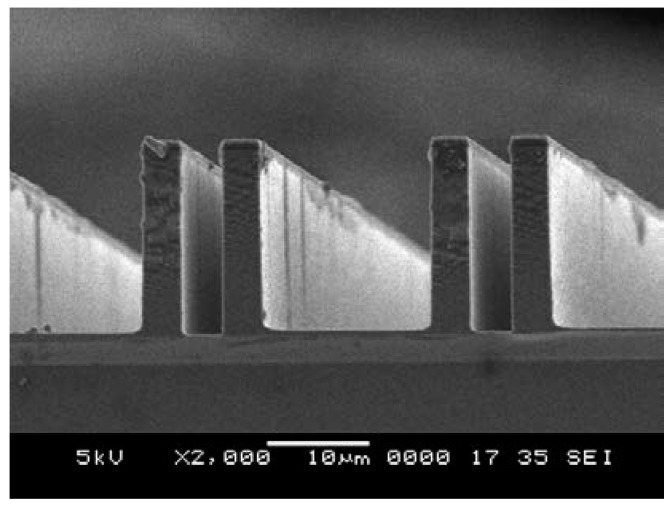
SEM image of the DRIE result after adjusting the process parameters.

Although the average etching rate is significantly slow (Table 2), reducing the lag effect is favorable (Figure 8).

## 3. Applications

### 3.1. Fabrication of Micro Gyroscope

**Figure 9:**
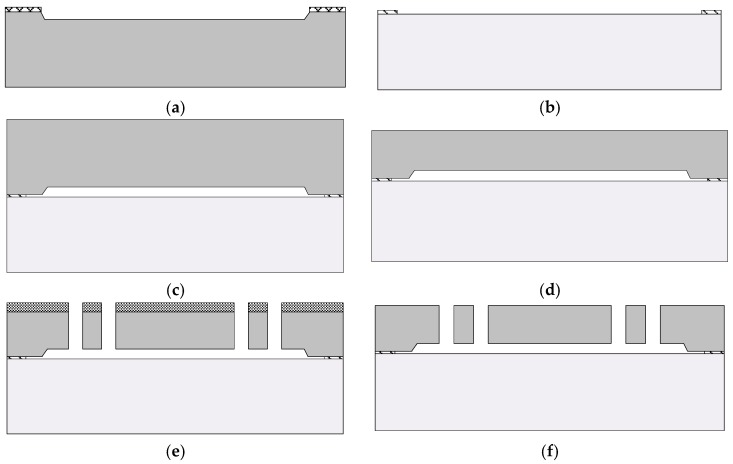
Silicon-on-glass (SOG) fabrication process used to implement the proposed gyroscope. (**a**) Formed anchors on silicon substrate; (**b**) Pattern of Cr/Au at the glass substrate; (**c**) Anodic bonding of silicon and glass substrates; (**d**) CMP of the silicon; (**e**) Pattern of the photoresist etch mask for DRIE and thoroughly etched silicon substrate; and, (**f**) Remove photoresist etch mask.

For confirming the feasibility of the modified CMP and DRIE processes, z-axis micro gyroscope with a fully symmetric structure is fabricated using the SOG process. The gyroscope sensor consists of the drive part and sense part. All of the fingers have the same geometric size, 100 μm in length, 5 μm in width, and 50 μm thick, and the gap of the fingers is 4 μm. The mass area of the sensor is 1840 μm × 1840 μm with 40 μm × 40 μm holes. The supporting beam around the mass is 500 μm in length and 10 μm in width. The SOG fabrication process is shown in Figure 9.

**Figure 10:**
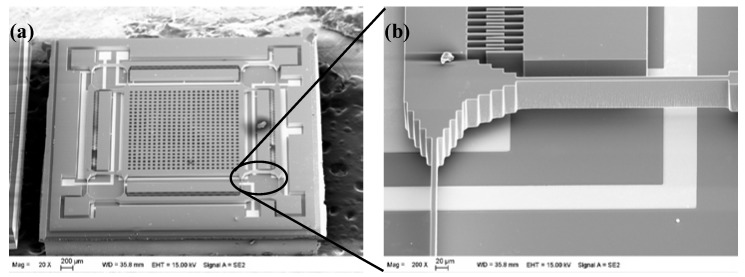
SEM of micro gyroscope fabricated by the SOG process. (**a**) Top view of the gyroscope; (**b**) Side view of the gyroscope.

First, the anchor regions are defined by wet etch on a 500 μm-thick silicon substrate to ensure the gap between the moving part and the substrate by use of the TMAH etchant. Then, 40 nm Cr and 100 nm Au are sputtered and patterned to form the electrodes on the Pyrex glass for signal line. Next, the silicon and Pyrex glass are bonded using the standard anodic bonding process. Thereafter, the silicon of the bonded wafer is lapped and polished using the mentioned process, and the designed thickness of the silicon is determined by the CMP process. Finally, the silicon structure is thoroughly etched using the modified DRIE process. Figure 10 shows the SEM image of the fabricated gyroscope. As shown in Figure 10b, no footing effect is observed, all of the structures are well defined. 

### 3.2. Test of the Fabricated Micro Gyroscope

**Figure 11:**
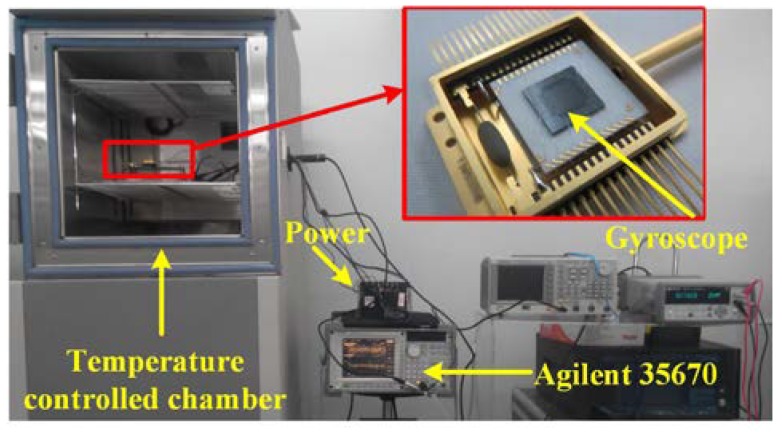
Test system of the gyroscope.

After fabricating the micro gyroscope, the resonant frequency of the sensor, including the drive mode and sense mode, has been tested simultaneously. The test system, as shown in Figure 11, is mainly composed of DC power, gyroscope with its interface circuits. A data acquisition unit (Agilent 35670A) is used to collect the output of gyroscope to obtain the resonant frequency.

**Figure 12:**
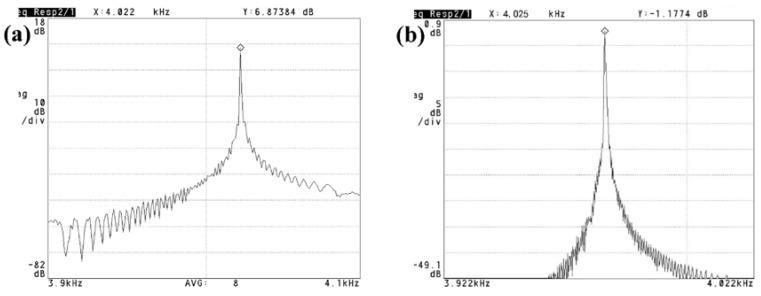
Resonant frequency and quality factor measurement. (**a**) Drive mode; (**b**) Sense mode.

As shown in Figure 12, the measured resonant frequencies of the drive and sense modes are 4.022 and 4.025 kHz, respectively. Frequency difference of these two modes is just 3 Hz, which means that the proposed fabrication method could achieve high mode match in order to improve the performance of the gyroscope. Further, after vacuum packaging, the quality factors of the drive mode and sense mode reach 12,058 and 10,437 by −3 dB measurement method, respectively.

**Figure 13:**
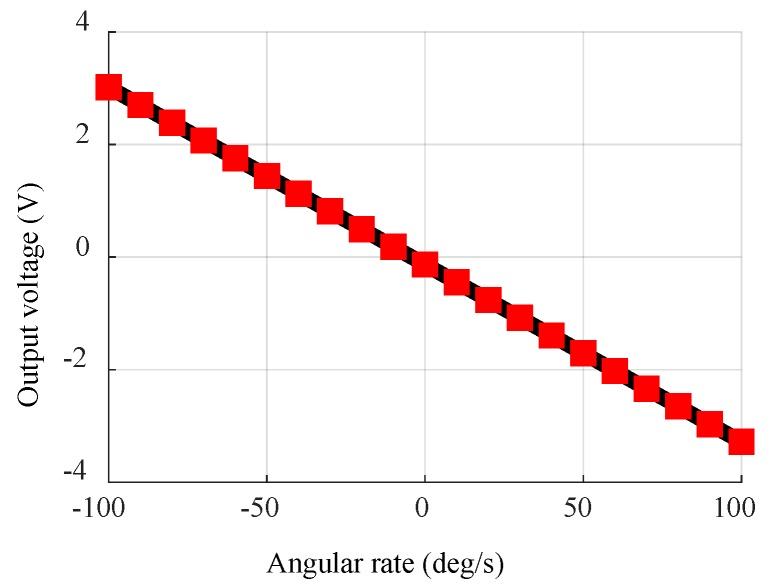
Scale factor curve.

The scale factor, noise density, and ARW of the gyroscopes are measured. The scale factors of the gyroscopes are measured by a rotational table. The Figure 13 shows the output of the gyroscope versus the applied angular rate from −100 deg/s to 100 deg/s with the scale factors of 32.5 mV/deg/s.

**Figure 14:**
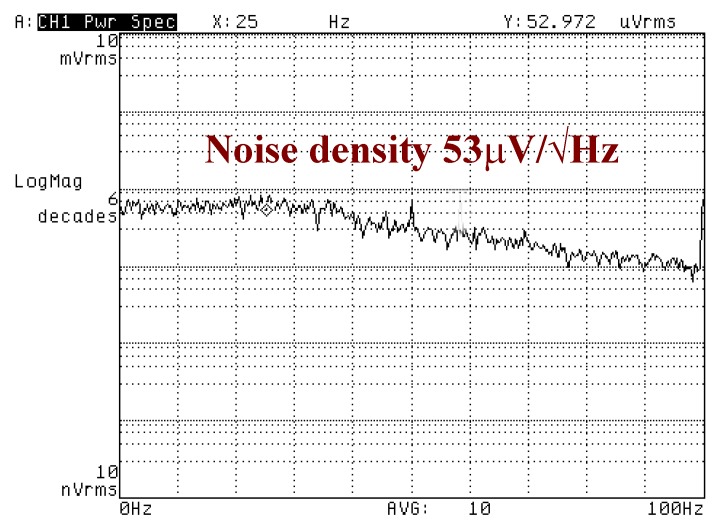
Equivalent angular rate.

Figure 14 shows the measured noise density plot of the gyroscopes. The calculated of the equivalent angular rate is about 1.6‰°/s/√Hz. Allan variance measurements is implemented with an ARW of 1.026°/√h. 

## 4. Conclusions

DRIE and CMP are the most important processes for the SOG process, and they are used to define the movable and the high-aspect-ratio silicon structures. Several damages in lapping process, such as breakthrough and the underlying structures at localized points that are caused by wafer-to-wafer misalignment during anodic bonding techniques, are mainly investigated. These damages can be solved by decreasing the load weight and increasing the polishing times. At the same time, the lag effect causes etch uniformity and difficulty in controlling etch endpoint and degrades the mechanical performance, thereby leading to device failure together and the footing effect. The main parameters, such as the flowrate of SF6, C4F8, oxygen, coil power, and platen power are adjusted to reduce the lag effect. As a result, the ratio of etching and passivation cycle time and the process pressure play important roles in the lag effect. The process is optimized to avoid the notching lag effects on the fabricated silicon structures during DRIE. Finally, a SOG z-axis gyroscope is fabricated for demonstrating the silicon removal of SOG wafer using the optimized DRIE and CMP processes. The initial test results show that the drive and sense modes of the micro gyroscope fabricated with the modified processes are 4.048 and 4.076 kHz, respectively. The demands for this kind of SOG MEMS device can be fulfilled by the use of the optimized processes.

## Figures and Tables

**Table 1:** Process parameters for chemical mechanical polishing (CMP).

Process	Powder	Thickness of the Silicon [μm]					Ra [nm]
		Center	Primary	Secondary	Left	Right	Average
Lapping	20 μm Al_2_O_3_	256	254	251	253	254	85.04
	9 μm Al_2_O_3_	204	204	204	204	203	79.29
Polishing	3 μm cerium oxide	202	203	203	202	203	8.19
	SF1	201	200	201	201	201	1.13

**Table 2:** Process Parameters of DRIE.

Recipe	Etching/Passivation (s)	Pressure (mTorr)	Average Etching Rate (μm/min)
a	6/5	43	4.67
b	8/10	35	3.23
c	8/10	30	2.56

**Table 3:** Optimal Parameters of DRIE.

Etch		Passivate	
SF_6_ flow rate (sccm)	180	C_4_F_8_ flow rate (sccm)	120
O_2_ flow rate (sccm)	20		
Coil power (w)	800	Coil power (w)	800
Platen power (w)	20	Platen power (w)	0
Cycle time (s)	8	Cycle time (s)	10
Pressure (mTorr)	30		
